# Mendelian randomization for studying the effects of perturbing drug targets

**DOI:** 10.12688/wellcomeopenres.16544.2

**Published:** 2021-02-10

**Authors:** Dipender Gill, Marios K. Georgakis, Venexia M. Walker, A. Floriaan Schmidt, Apostolos Gkatzionis, Daniel F. Freitag, Chris Finan, Aroon D. Hingorani, Joanna M.M. Howson, Stephen Burgess, Daniel I. Swerdlow, George Davey Smith, Michael V. Holmes, Martin Dichgans, Robert A Scott, Jie Zheng, Bruce M. Psaty, Neil M. Davies

**Affiliations:** 1 Department of Epidemiology and Biostatistics, School of Public Health, Imperial College London, London, UK; 2 Centre for Pharmacology and Therapeutics, Department of Medicine, Imperial College London, London, UK; 3 Novo Nordisk Research Centre, Oxford, UK; 4 Clinical Pharmacology and Therapeutics Section, Institute of Medical and Biomedical Education and Institute for Infection and Immunity, St George’s, University of London, London, UK; 5 Clinical Pharmacology Group, Pharmacy and Medicines Directorate, St George’s University Hospitals NHS Foundation Trust, London, UK; 6 Institute for Stroke and Dementia Research (ISD), University Hospital of Ludwig-Maximilians-University (LMU), Munich, Germany; 7 Medical Research Council Integrative Epidemiology Unit, University of Bristol, Bristol, UK; 8 Population Health Sciences, Bristol Medical School, University of Bristol, Bristol, UK; 9 Department of Surgery, Perelman School of Medicine, University of Pennsylvania, Philadelphia, PA, USA; 10 Institute of Cardiovascular Science, Faculty of Population Health, University College London, London, UK; 11 Department of Cardiology, Division Heart and Lungs, University Medical Center Utrecht, Utrecht, The Netherlands; 12 Medical Research Council Biostatistics Unit, University of Cambridge, Cambridge, UK; 13 Bayer Pharmaceuticals, Open Innovation & Digital Technologies, Wuppertal, Germany; 14 UCL British Heart Foundation Research Acceleratorversity College London, London, UK; 15 UCL Hospitals, NIHR Biomedical Research Centre, London, UK; 16 Cardiovascular Epidemiology Unit, Department of Public Health and Primary Care, University of Cambridge, Cambridge, UK; 17 NIHR Bristol Biomedical Research Centre, University of Bristol, Bristol, UK; 18 Medical Research Council Population Health Research Unit, University of Oxford, Oxford, UK; 19 Munich Cluster for Systems Neurology (SyNergy), Munich, Germany; 20 German Centre for Neurodegenerative Diseases (DZNE), Munich, Germany; 21 Human Genetics, GlaxoSmithKline, Stevenage, UK; 22 Cardiovascular Health Research Unit, Departments of Medicine, Epidemiology and Health Services, University of Washington, Seattle, WA, USA; 23 Kaiser Permanente Washington Health Research Institute, Seattle, WA, USA; 24 K.G. Jebsen Center for Genetic Epidemiology, Department of Public Health and Nursing, NTNU, Norwegian University of Science and Technology, Trondheim, Norway

**Keywords:** Drugs, Genetics, Mendelian randomization

## Abstract

Drugs whose targets have genetic evidence to support efficacy and safety are more likely to be approved after clinical development. In this paper, we provide an overview of how natural sequence variation in the genes that encode drug targets can be used in Mendelian randomization analyses to offer insight into mechanism-based efficacy and adverse effects. Large databases of summary level genetic association data are increasingly available and can be leveraged to identify and validate variants that serve as proxies for drug target perturbation. As with all empirical research, Mendelian randomization has limitations including genetic confounding, its consideration of lifelong effects, and issues related to heterogeneity across different tissues and populations. When appropriately applied, Mendelian randomization provides a useful empirical framework for using population level data to improve the success rates of the drug development pipeline.

## Introduction

The majority of small molecule and biologic drugs exert their effects by perturbing protein targets ^1^. The identification of such targets is therefore central to drug discovery. Despite increasing investment in research and development within the pharmaceutical industry ^2^, overall drug development failure rates remain high ^3– 8^, most notably for targets that represent novel mechanisms. Such failures result in increased costs and reduced availability of novel agents ^9^.

With the recent growth in genetic data ^10^, there has been substantial progress in the identification of genes that are linked to human health and disease. Genetic data can potentially be used for identifying and prioritizing novel drug targets and indications ^2^. For example, genome-wide association studies (GWAS) have corroborated approximately 70 of the 670 known effects of licensed drugs through associations at the loci of the genes coding for their corresponding target proteins ^11^. Studies of drug development programs have also shown that targets with genomic support have a higher rate of success ^2, 12– 15^.

### Mendelian randomization

Through the random allocation of genetic variants at conception, genetic studies in human populations can imitate the design of randomized controlled trials (RCT) ^16, 17^. Such investigation uses genetic variants as instrumental variables for studying the effect of an exposure on an outcome, and has been referred to as Mendelian randomization (MR) ^18^. Phenotypic observational studies are limited in their ability to draw causal inferences due to bias from confounding and reverse causation ^18^. In contrast, MR uses the random allocation of genetic variants from parents to offspring during conception to guard against these biases.

MR requires the following instrumental variable assumptions: the genetic variant i) is associated with the exposure (relevance), ii) has no common cause with the outcome (independence), and iii) only affects the outcome via the exposure (exclusion restriction) ^19, 20^. The first of these is testable; the remaining assumptions are untestable but falsifiable. Assumption iii) the exclusion restriction, assumes that the genetic variant affects the outcome through the exposure and not any other horizontally pleiotropic pathways ^18, 21^. Further assumptions are also required to obtain valid point estimates, for instance, that the influence of the exposure on the outcome is the same for all individuals (effect homogeneity) or that the exposure is a monotonic (always increasing or always decreasing) function of the instrument for all individuals in the population (monotonicity) ^19^. In addition, the interpretation of MR findings can have particular nuances, as previously described ^22^.

**Figure 1.**
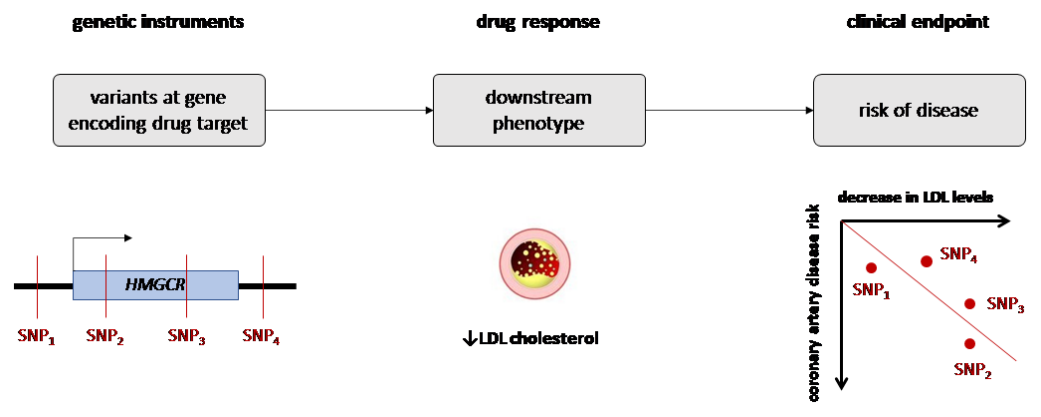
Principles of Mendelian randomization studies (MR) studying drug effects. MR makes use of genetic variants located within or close to a gene encoding a drug target (e.g. at *HMGCR* encoding the drug target of statins) that lead to downstream effects similar to the desired drug response (e.g. lowering of low-density lipoprotein [LDL] cholesterol) in order to explore effects on clinical outcomes (e.g. risk of coronary artery disease). SNP: single-nucleotide polymorphism.

Where the exposure under study is perturbation of a drug target, MR can be used to explore drug effects ( Figure 1) ^23, 24^. For drug target MR specifically, genetic variants such as single-nucleotide polymorphisms (SNPs) related to the function or expression of the drug target protein can be used as instrumental variables to study the effect of perturbing that drug target ^25, 26^. These variants are typically in or near the gene that encodes the drug target ( *cis*-variants). Such MR can be used in drug development to investigate the likely efficacy and safety of perturbing novel drug targets ^27, 28^, as well as explore the repurposing potential and adverse effects of existing drugs ^25^.

The identification and validation of appropriate genetic variants as instrumental variables for an exposure is critical for the design and interpretation of all MR analyses ^29^. While previous work has offered practical advice on selecting instruments for MR studies considering disease biomarkers ^24^, the field is continuing to evolve rapidly ^30^. The growth in genetic association study data that extends to tissue-specific gene expression ^31^, circulating proteins ^32^, metabolites ^33, 34^ and cytokines ^35^, has been coupled with increased efficiency of MR studies using automated software, databases, statistical packages and readily available code ^30, 36– 38^. However, there is still no consensus on the strategy for identifying genetic instruments and exploring potential drug effects with MR. Here we discuss practical considerations while also offering illustrative examples for the most relevant points. We describe issues relating to selection of genetic variants as proxies for drug target perturbation, evaluation of the plausibility of genetic variants as proxies for drug target perturbation, generation and interpretation of MR estimates, and limitations of MR for investigating drug target perturbation. Finally, we offer a step-by-step framework for how to conduct a drug target MR study ( Box 1).

**Box 1. Step-by-step guide for conducting Mendelian randomization (MR) analyses of drug target perturbation**

1. Determine the drug targets of interest
2. Identify the gene(s) encoding the relevant protein(s)
3. Choose data source for identifying instruments
4. Select genetic variants as instruments based on:
  a. Strength of associations with downstream effects of drug target perturbation
  b. Linkage disequilibrium structure
  c. Distance from gene(s) encoding the drug target
5. Validate genetic variants for use as instruments by confirming that they recapitulate known on-target drug effects
6. Estimate effects of drug target perturbation on outcome(s) of interest using MR
  a. Use appropriate method to account for linkage disequilibrium structure between variants
  b. Scale estimates appropriately
  c. Interpret MR as representing effects of lifelong drug target perturbation
7. Investigate potential adverse effects and repurposing opportunity using phenome-wide association study
8. Triangulate using other interventional, observational and experimental data

## Instrument selection

MR investigations of drug effects have mainly studied small molecule, peptide and biotherapeutic drugs ^39, 40^, where genetic instruments are selected as variants that mimic perturbation of their protein targets. Instrument selection can be considered in two parts: i) identifying the gene or group of genes corresponding to the drug target proteins and ii) selecting genetic variants to proxy perturbation of the drug targets. These steps are discussed in detail below, followed by consideration of drugs that have targets made up of multiple proteins.

### Identifying genes corresponding to drug target proteins

The key difference between conventional MR for an exposure and MR for the investigation of drug effects is that for the latter the instrument can be constructed in relation to the gene corresponding to the drug target, rather than genetic variants from across the genome ( Table 1). The first step of this process is therefore to identify the drug target of interest and its corresponding gene. Resources such as DrugBank (which is freely available for non-commercial purposes) provide information about existing drugs, including their mechanism of action, targets and their corresponding gene(s), and indications ^41^. Where the target of a drug is known, information regarding the corresponding gene can also be obtained from other databases such as Ensembl and UniProt ^42, 43^.

**Table 1.** Differences between conventional Mendelian randomization (MR) and MR specifically exploring drug target perturbation.

	Conventional MR	MR investigating drug effects
**Aim of the analysis**	To investigate the effect of an exposure on an outcome	To investigate the effect of perturbing a drug target on an outcome
**Genomic location of ** **instruments**	Genome-wide	Often restricted to the locus of the gene encoding the drug target under study
**Selection of genetic** **instruments**	Variants associated with the exposure under study	Variants associated with perturbation of the drug target under study
**Statistical analysis**	Typically uses uncorrelated variants; higher risk of pleiotropic effects on the outcome through pathways unrelated to the exposure	More frequent use of methods to account for correlation between instrument variants; lower risk of pleiotropic effects on the outcome through pathways unrelated to the drug target

### Selecting genetic variants to proxy drug targets

**Figure 2.**
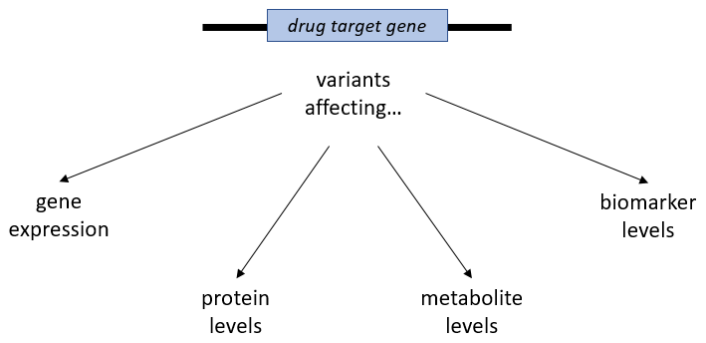
Potential strategies for selecting genetic variants as instruments for a protein drug target. Variants within or close to the drug target gene might be selected on the basis of their associations with gene expression and levels of the target protein or known downstream functions such as effects on metabolite levels or biomarkers. Notably, variants influencing gene expression and protein levels do not always influence the function of the derived protein product and might not translate to downstream effects comparable to those achieved by the pharmacological modulation of the drug target.

Several factors need to be considered when selecting genetic variants to proxy the effects of drug target perturbation. If MR is being used to investigate effects of perturbing the target of a drug with an existing indication, then instruments can be selected based on their location at the corresponding gene and association with that indication. For instance, Gill *et al.* selected genetic variants to proxy antihypertensive drug class effects as those located at the gene corresponding to the drug target that also related to systolic blood pressure in a GWAS ^44^. If the indication is not known, one possible approach is to use quantitative trait loci for expression of the gene encoding the drug target of interest (in relevant tissues or cell contexts) as instruments for drug target perturbation. An important limitation of gene expression is that variants affecting gene expression may not necessarily also affect protein expression, and *vice versa* ( Figure 2) ^45^. Furthermore, gene expression quantitative loci have been reported to account for little of the heritability of complex diseases ^46^. Therefore, protein expression quantitative loci may make better instruments for proxying drug effects than gene expression data, if they are available in relevant tissues and contexts.

As mentioned above, selected instruments for drug target perturbation are often restricted to *cis*-acting genetic variants – those in or close to the gene of interest. In general, such variants are more likely to have effects specific to the protein of interest than genetic variants that are not located within the gene locus ( *trans*-acting). This point is well highlighted by the example of C-reactive protein, for which MR analyses investigating effects of its circulating levels produce very different results depending on whether the instruments are selected from the *CRP* locus or from throughout the genome ^24^. Related considerations include how proximal *cis*-variants should be to the corresponding gene, and whether enhancer or promoter regions for the gene should be counted as cis-acting ^47^. While some evidence currently supports that genetic variants affecting gene expression typically lie within 200kB of the gene locus ^48^, there is no established consensus on the issue of proximity. The pertinent requirement is that the variant be related to the function or expression of the drug target. In the past, single-region MR analyses have sometimes used only the top variant in the region as a genetic instrument ^28, 49, 50^. However, this approach can also be suboptimal if studying a region containing variants that have multiple conditionally independent associations with the exposure. For example, genetic association studies have suggested that the *SHBG* region encoding sex-hormone binding globulin (SHBG) may harbor up to nine variants independently associated with circulating SHBG concentration ^51^, and that using only the top variant may limit the statistical power of such MR analysis.

The degree to which variants at the same locus should be allowed to correlate with each other through linkage disequilibrium (LD) while still being modelled as independent also warrants attention. Unaccounted correlation between the variants used can result in underestimation of the standard error of MR estimates, yet there is no recommended LD threshold. To circumvent this issue, methods are available to adjust for LD between genetic variants used as instruments, which may help confirm the robustness of the findings and maximize statistical power ^52– 54^.

### Investigating drugs with multiple targets

Many drugs do not have a target that is encoded by a single gene. For example, the calcium channel blocker class of antihypertensive drugs have targets that are made up of proteins coded by several different genes ^44, 55^. At present there is no consensus on the best way to combine data from multiple genes corresponding to a single target into an instrument. Previous studies have selected genetic variants related to the individual genes and combined their data to investigate the effect of perturbing the drug target, while applying clumping to ensure independence as described above ^44, 55^.

## Instrument evaluation

Once the instrument has been selected, it can be evaluated to ascertain its validity for the analysis of interest. MR analyses exploring drug effects can be biased if the genetic variants incorporated as instruments have “horizontal” pleiotropic effects, where there are pathways from the variant to the specific outcome under consideration that do not pass through the exposure of interest ^56^. In contrast, “vertical” pleiotropy lies on the causal pathway between the pharmacological mechanism and outcome ^56^. Vertical pleiotropy does not bias MR estimates and is often of interest as it can provide insight into causal mechanisms and mediation. As with MR generally, one of the most useful approaches for evaluating instrument validity is to investigate its relation to a known effect of the exposure under consideration ^57^. This approach is feasible for MR used to predict the effect of perturbing targets for which there are drugs with established indications and known associations with biomarkers ^58^. For example, Walker *et al.* selected genetic variants to proxy antihypertensive drugs from gene expression data and validated these instruments through their associations with systolic blood pressure, prior to applying MR analyses investigating the outcome of interest, Alzheimer’s disease ^55^. An instrument may also be examined in relation to potential confounders, in order to investigate violations of the independence and exclusion restriction assumptions necessary for MR ^56^. Berry *et al.* illustrated such an approach during their evaluation of genetic proxies for vitamin D status ^59^. In this study, the association of variants with social, dietary and lifestyle factors was investigated, to identify potential sources of confounding. 

Complementary data may also be used for instrument evaluation. For example, MR studies designed to investigate the effect of genetically predicted variations in interleukin-6 (IL6) signaling would be expected to show that the selected instruments associate with molecules that are downstream of the pathway ^49^. Genetic association estimates for the serum levels of several of these molecules are available, including IL6 and IL6 receptor (IL6R), C-reactive protein (CRP) and fibrinogen ^60^. Hence, if the selected genetic instruments are valid proxies for IL6 signaling, they may be expected to show consistent effects across these molecules. An alternative example is provided by Wurtz *et al.* who demonstrated consistency between the metabolic changes associated with starting statins and metabolomic associations of the *HMGCR* variant rs12916 that was used to proxy statin effect ^61^.

## Analysis

Given a set of genetic instruments, the statistical methods used for MR investigation of drug target perturbation are similar to those used for MR more generally ^62^. Interpretability is often facilitated by scaling of genetic associations to unit change in a trait related to drug target perturbation. For example, for analyses considering associations of variants in the *HMGCR* gene that are used to proxy statin drug effects, estimates may be scaled to change in low-density lipoprotein cholesterol levels ^50, 63– 65^. As another example, for analyses investigating IL6R signaling using variants in the *IL6R* gene, effects may be scaled to downstream changes in CRP levels ^49, 60^. Care must be taken in the interpretation of such scaled estimates however, because although MR estimates may be directionally concordant to the effect of drug target perturbation on the biomarker, their magnitudes may not be comparable ^66^.

Statistical approaches used to evaluate potential bias from horizontal pleiotropy in MR analyses can also be used in MR investigating drug target perturbation ^62^. However, variants selected as instruments for drug target perturbation are often selected from within a specific locus rather than from throughout the genome, and may be limited in number. Statistical sensitivity analyses for investigating horizontal pleiotropy typically require large numbers of genetic variants, and so may not be suitable for many drug target MR analyses ^29^. Assessment of heterogeneity between MR estimates produced by variants in a single locus is still possible however, and can be used to inform on potential bias related to horizontal pleiotropy ^53, 67^.

In an effort to better explore the target region and increase statistical power, genetic variants that have weaker associations with perturbation of the drug target may be considered as instruments ^27, 68^. Despite the potential benefits of this approach ^69^, care must be taken to avoid weak instrument bias ^54, 70^. Under a two-sample design, weak instrument bias will attenuate MR estimates towards the null ^71^.

MR can be used to assess a wide range of outcome traits and thus investigate potential effects of perturbing the drug target on these traits ^72^. Such studies are often conducted as hypothesis-free, phenome-wide association analyses (PheWAS) ^73, 74^, and can be helpful for exploring potential adverse effects or identifying previously unknown re-purposing opportunities. For example, Schmidt *et al.* conducted a PheWAS of the *PCSK9* locus to assess potential adverse effects of PCSK9 inhibitor drugs ^75^.

In addition to using MR, it is also possible to generate genetic evidence supporting a causal effect of drug target perturbation on an outcome by identifying proportionality of genetic associations with traits proxying drug target perturbation and the outcome, at the corresponding drug target gene locus. Such investigation is referred to as genetic colocalization, and can help distinguish causation from genetic confounding (such as may arise due to horizontal pleiotropy). Popular colocalization methods include coloc ^76^, moloc ^77^, eCAVIAR ^78^ and HEIDI ^79^. However, a limitation of many colocalization approaches is that they assume there is only a single causal variant at the considered locus.

Triangulation, the practice of integrating evidence from several different methodological approaches and data sources that each differ in their susceptibility to bias, is another important aspect of interpreting the analysis ^80^. MR evidence should be considered alongside other study designs to increase confidence in findings ^58^. For example, the European Atherosclerosis Society consensus statement on the role of low-density lipoproteins on atherosclerotic cardiovascular disease considers evidence from inherited disorders of lipid metabolism, prospective epidemiologic studies, MR investigations and RCTs ^81^. Moreover, comparing instruments between different MR studies of the same exposure can provide additional evidence. For example, both Gill *et al.* and Walker *et al.* independently derived instruments for antihypertensive drug effects that perform comparably when tested against a common outcome ^44, 55^. Although different MR studies may use similar or overlapping data sources, different instrument selection approaches can make analyses vulnerable to distinct biases and so also have a role in triangulation of evidence.

## Limitations

**Figure 3.**
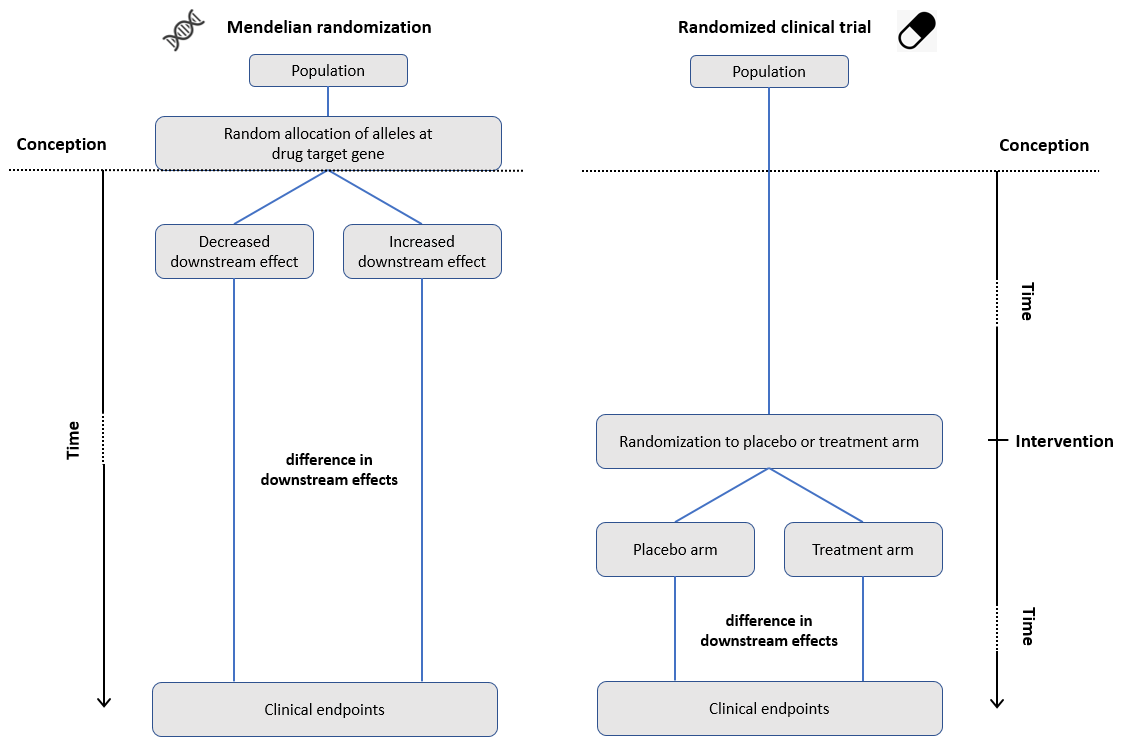
Comparison between Mendelian randomization (MR) study for drug effects and randomized clinical trial (RCT). Similar to the randomization process of RCTs, the random allocation of alleles at a drug target gene in MR studies allows the distribution of individuals to groups that differ only regarding the downstream effects of the drug target and not other confounders. While the random allocation of alleles in MR studies happens at conception and leads to lifelong effects, the randomization in RCTs typically happens later in life and focuses on the effects of short-term interventions.

As with all research methods, MR has limitations ^82^. RCTs remain the best source of evidence evaluating drug efficacy and guiding clinical practice ^83^. While MR and RCTs have the same aim – reliable evidence of causation – they estimate different treatment parameters, which are not directly comparable. Genetic variants typically have smaller effects which accumulate across the entire life-course, whereas pharmacological agents are often prescribed later in life and typically have larger effects. Therefore, MR estimates reflect the lifelong effects of perturbing a drug target, which may not be equivalent to interventions given at a specific point in time and for a shorter time period ( Figure 3). While these differences make it unlikely that MR estimates will accurately reflect the size of effect of a pharmacological intervention, they are still a useful indication of presence and direction of causal effects ^58^.

A further limitation of MR for studying the effects of drug target perturbation is that it may not account for post-transcriptional and post-translational modification in the pathway from a gene to a biologically functional protein. Well-conducted MR analyses may be able to inform broadly on drug class effects, but not necessarily provide information on the effects of a specific pharmacological agent. For example, dihydropyridine and non-dihydropyridine subclasses of calcium-channel blocker antihypertensive drugs have distinct pharmacological effects. Genetic variants that affect blood pressure via calcium-channel blockade can estimate the effects of calcium-channel targeting drugs in general, but cannot differentiate the relative effects of dihydropyridine versus non-dihydropyridine subclasses ^44^. Furthermore, MR in this context is applied to drug targets and not compounds – so it can be used to investigate the effects of perturbing a drug target, but is unlikely to be able to offer insight towards molecule specific effects ^28^.

Drug effects also vary in different tissues and populations, and similarly MR estimates for the effects of perturbing drug targets may only be valid if genetic association data from the relevant tissues or populations are used. This limitation can have implications for both identifying instruments and using MR to study drug effects, as highlighted in an example that used gene expression data to identify instruments for antihypertensive drug classes in the investigation of repurposing potential for the prevention of Alzheimer’s disease ^55^. Here, it is not clear whether the same genetic variants related to gene expression in vascular, cardiac and brain tissue. Furthermore, to date, most genotyped samples have been sampled from European ancestry populations. While this approach minimizes the risk of population stratification and false-positive GWAS signals, consideration of distinct ancestral groups is likely to offer novel insight. For example, genetic evidence on the effects of alcohol comes from variants in the *ALDH2* gene, which are common in Asian, but not European populations ^84^.

## Conclusion

Over the last decade, MR has become a widely used epidemiological tool for estimating the causal effects of risk factors on clinical outcomes. On top of this well-studied application, there are now multiple examples highlighting its power for investigating drug effects. Despite its explicit assumptions, modern developments in statistical methodology and the widespread availability of multiple levels of omics data have provided the necessary resources to more reliably and efficiently use MR in order to study drug effects. As such, it has found a growing niche within the broader framework for exploring therapeutic targets, efficacy, adverse effects and repurposing potential. Given the high failure rates of clinical trials and that drug targets with genetic support are more likely to make it through the development pipeline ^13, 15, 23^, MR can provide evidence for prioritizing agents to move forward in development.

## Data availability

No data is associated with this article.
