# Supplementary material for: Mendelian randomization for studying the effects of perturbing drug targets
Source: Wellcome Open Res. 2021 Feb 10;6:16. Originally published 2021 Jan 28. [Version 2] doi: 10.12688/wellcomeopenres.16544.2 (PMC7903200; doi:10.12688/wellcomeopenres.16544.2)
Supplement: Peer review history

# Peer review: Reviewer response for version 2

**DOI:** 10.21956/wellcomeopenres.18307.r42576

**Published:** 2021-03-01

**Authors:** Matthew Traylor

**Affiliations:** 1 Clinical Pharmacology, William Harvey Research Institute, Queen Mary University of London, London, UK

This is a clear and timely review on the use of Mendelian Randomization for studying the effects of perturbing drug targets.

 I have some minor comments for the authors' consideration:

 In the discussion of 'selecting genetic variants to proxy drug targets', the authors discuss using instruments based on location at a corresponding gene. Although this approach is likely a valid one in many instances, there are exceptions where trans effects could confound associations. An example is the FTO association with BMI where variants close to the FTO gene have no impact on FTO and instead influence IRK3 / IRK5 expression via trans effects ( https://pubmed.ncbi.nlm.nih.gov/26287746/). ^1^ I did therefore wonder if some discussion of the potential of this approach to be susceptible to failures of the relevance assumption of MR might be merited.

 Similarly, I did wonder if more expanded discussion of the relative merits of the use of proteomics compared to transcriptomics, as discussed in recent work (https://pubmed.ncbi.nlm.nih.gov/32591531/) ^2^ could be included - as well as discussion of the importance of tissue specificity.

 The section under analysis discussing colocalization approaches should be updated to reflect the latest publications, which offer more flexibility in terms of assumptions of numbers of causal variants ( https://www.biorxiv.org/content/10.1101/2021.02.23.432421v1). ^3^

Is the review written in accessible language?

Yes

Are all factual statements correct and adequately supported by citations?

Yes

Are the conclusions drawn appropriate in the context of the current research literature?

Yes

Is the topic of the review discussed comprehensively in the context of the current literature?

Yes

Reviewer Expertise:

Statistical Genetics

I confirm that I have read this submission and believe that I have an appropriate level of expertise to confirm that it is of an acceptable scientific standard.

## References

[1] : FTO Obesity Variant Circuitry and Adipocyte Browning in Humans. *N Engl J Med*.2015;373(10) : 10.1056/NEJMoa1502214 895-907 10.1056/NEJMoa1502214 26287746PMC4959911

[2] : Genetic drug target validation using Mendelian randomisation. *Nature Communications*.2020;11(1) : 10.1038/s41467-020-16969-0 10.1038/s41467-020-16969-0 PMC732001032591531

[3] : A more accurate method for colocalisation analysis allowing for multiple causal variants. *bioRxiv*.2021; 10.1101/2021.02.23.432421 10.1101/2021.02.23.432421 PMC850472634587156

# Peer review: Reviewer response for version 2

**DOI:** 10.21956/wellcomeopenres.18307.r42580

**Published:** 2021-02-25

**Authors:** C. Mary Schooling

**Affiliations:** 1 School of Public Health and Health Policy, City University of New York, New York, NY, USA

Mendelian randomization (MR) is a fantastic method that has revolutionized our understanding of risk factors for many diseases, and whose further use in identifying drug targets is immensely valuable. This is a helpful guide to using Mendelian randomization (MR) to investigate drug targets but needs to address some additional relevant issues that could confuse researchers.

 Key points that need to be made clearer:

1. MR does not quite imitate a randomized controlled trial (RCT). RCTs randomize to the drug or intervention after recruitment, meaning well-conducted RCTs are not open to confounding (bias typically from common causes of intervention/exposure and outcome) or selection bias (typically from selecting on exposure and outcome). MR studies usually use genetic studies recruited in mid-life well after the randomization of genetic material at conception. As such, MR studies usually only concern survivors of their genetic make-up and also of the disease of interest, i.e., they may be selected on genetic variants and the disease of interest, meaning they are open to selection bias. In this situation estimates for the drug target on the disease of interest might be attenuated, reversed, or spurious. In addition, inadvertently selecting survivors of their genetic variants and competing risk of the disease of interest will similarly affect the estimate for the drug target on the disease of interest, even if nobody has died of the disease of interest before recruitment. Examples of this issue include the genetic variants corresponding to PCSK9 inhibitors appearing to have no effect on stroke ^1^ and the genetic variant corresponding to statins, i.e., rs12916, appearing to have no effect on stroke. ^2^ These findings conflict with RCTs, and are likely the result of selection bias, as described above.
2. Unlike RCTs, MR is best suited for investigations of the effects of drugs intended to reduce the risk of disease but is not necessarily suitable for studies of drug effects in patients. An MR study of a disease treatment drug in patients would be open to selection bias because patients are typically survivors of their genetic make-up and of the disease of interest. This point has been explained in relation to genetic studies, ^3^ and solutions are under-development for both genetic studies and MR. Researchers often want to use MR to assess effects of drugs in a patient sample just as they can in an RCT, so it needs to be explained when such an MR study would and would not be likely to give valid results.
3. Criteria for suitable samples for selecting genetic instruments for a drug need to be given. For example, should the sample be population-representative, in young people, from a trial of the relevant drug, from controls only of a case-control study?

There are also some other points that need clarification.

1. Paragraph 3, The major sources of bias in phenotypic observational studies are now understood to be confounding and selection bias, as explained here. ^4^^,^^5^ So, selection bias needs to be added to the second to last sentence of this paragraph. The last sentence of the same paragraph then needs to be amended slightly, because MR is open to selection bias, as explained above.
2. Paragraph 4, the elaboration of the exclusion restriction should explain what “horizontal pleiotropy” is because it has not been mentioned before. Please also make it clear that this assumption is violated if any open path from genetic variant to outcome exists that does not affect the exposure. ^6^ Instrument evaluation first paragraph, please similarly clarify the first sentence.
3. In the Limitations section, or elsewhere, please explain the differences between an RCT and an MR study in terms of selection bias and types of questions that can be answered by each design. Ensure Figure 3 reflects these differences.

Minor points:

1. Please avoid jargon, such as “clumping”.

Is the review written in accessible language?

Yes

Are all factual statements correct and adequately supported by citations?

Partly

Are the conclusions drawn appropriate in the context of the current research literature?

Partly

Is the topic of the review discussed comprehensively in the context of the current literature?

Yes

Reviewer Expertise:

Epidemiological methods, drug effects, implications of the trade-off between reproduction and longevity for public health interventions

I confirm that I have read this submission and believe that I have an appropriate level of expertise to confirm that it is of an acceptable scientific standard, however I have significant reservations, as outlined above.

## References

[1] : Differential effects of PCSK9 variants on risk of coronary disease and ischaemic stroke. *European Heart Journal*.2018;39(5) : 10.1093/eurheartj/ehx373 354-359 10.1093/eurheartj/ehx373 PMC583748929020353

[2] : Use of Multivariable Mendelian Randomization to Address Biases Due to Competing Risk Before Recruitment. *Front Genet*.2020;11: 10.3389/fgene.2020.610852 610852 10.3389/fgene.2020.610852 33519914PMC7845663

[3] : Adjustment for index event bias in genome-wide association studies of subsequent events. *Nature Communications*.2019;10(1) : 10.1038/s41467-019-09381-w 10.1038/s41467-019-09381-w PMC645090330952951

[4] : A structural approach to selection bias. *Epidemiology*.2004;15(5) : 10.1097/01.ede.0000135174.63482.43 615-25 10.1097/01.ede.0000135174.63482.43 15308962

[5] : Causal inference and the data-fusion problem. *Proceedings of the National Academy of Sciences*.2016;113(27) : 10.1073/pnas.1510507113 7345-7352 10.1073/pnas.1510507113 PMC494150427382148

[6] : Causality: models, reasoning, and inference. *Cambridge University Press*.2009;

# Peer review: Reviewer response for version 2

**DOI:** 10.21956/wellcomeopenres.18307.r42579

**Published:** 2021-02-23

**Authors:** Shiu Lun Ryan Au Yeung

**Affiliations:** 1 School of Public Health, Li Ka Shing Faculty of Medicine, The University of Hong Kong, Hong Kong, Hong Kong

Review of the manuscript (Gill *et al.*, 2021)

 This is a clearly written paper concerning the use of genetics to infer drug’s effects on health outcomes. The paper summarises landmark studies/commentaries in this area of research and have covered the majority of the points. I have some comments for the authors’ consideration.

**Major comments**

- Although the use of genetics to infer effect of medication is promising, one of the challenges is the identification of a relevant variant to mimic medication use. Previous papers mainly use postulated phenotypes to identify the relevant causal variant (e.g. LDL cholesterol for HMGCR or systolic blood pressure for genetic targets of anti-hypertensives). However, the choice may not be that clear for other medications. For example, researchers may choose HbA1c over fasting glucose (PMID: 32748028) ^1^ to identify variants related to metformin because of sample size consideration and genotype coverage in the phenotype datasets (as per MAGIC database). Similarly, there may be pleiotropic effects of medication that are not mediated via the proposed phenotypes used to select the variants in Mendelian randomization and hence may not be able to capture the total effect of the medications. I think these are challenges that would be worth discussing to stimulate additional research. The use of proteomic data may help solve the abovementioned problem although proteomic datasets may not be as large and hence may be underpowered to identify relevant variants.
- Can the authors also comment on other approaches such as factorial MR design to investigate combined effects of medications (PMID: 27959767)? ^2^
- Although Mendelian randomization studies are less vulnerable to confounding than observational studies, selection bias could still be an issue, in particular when this method is used to explore potential targets for diseases that have a late on-set year (e.g. Alzheimer’s Disease).
- I think another challenge would be to investigate the effect of medication amongst patients (PMID: 28981501). ^3^
- I am not sure if sensitivity analyses for correlated variants perform as well as uncorrelated variants. For example, a previous study commented that although MR-Egger can be adapted to correlated variants, the underlying assumption is unlikely met (PMID: 28527048). ^4^

**Minor comments:**

- Figure 3: I think it should be randomized controlled trial for RCT.
- For reference 26, I think this is now being published and can be updated ( https://www.nature.com/articles/s41467-020-16969-0)

Is the review written in accessible language?

Yes

Are all factual statements correct and adequately supported by citations?

Yes

Are the conclusions drawn appropriate in the context of the current research literature?

Yes

Is the topic of the review discussed comprehensively in the context of the current literature?

Yes

Reviewer Expertise:

Cardiovascular diseases; Diabetes; Mendelian randomization

I confirm that I have read this submission and believe that I have an appropriate level of expertise to confirm that it is of an acceptable scientific standard.

## References

[1] : Evaluating the impact of AMPK activation, a target of metformin, on risk of cardiovascular diseases and cancer in the UK Biobank: a Mendelian randomisation study. *Diabetologia*.63(11) : 10.1007/s00125-020-05243-z 2349-2358 10.1007/s00125-020-05243-z 32748028

[2] : Variation inPCSK9 andHMGCR and Risk of Cardiovascular Disease and Diabetes. *New England Journal of Medicine*.2016;375(22) : 10.1056/NEJMoa1604304 2144-2153 10.1056/NEJMoa1604304 27959767

[3] : Genetic epidemiology and Mendelian randomization for informing disease therapeutics: Conceptual and methodological challenges. *PLoS Genet*.2017;13(10) : 10.1371/journal.pgen.1006944 e1006944 10.1371/journal.pgen.1006944 28981501PMC5628782

[4] : Interpreting findings from Mendelian randomization using the MR-Egger method. *Eur J Epidemiol*.32(5) : 10.1007/s10654-017-0255-x 377-389 10.1007/s10654-017-0255-x 28527048PMC5506233

[5] : Genetic drug target validation using Mendelian randomisation. *Nature Communications*.2020;11(1) : 10.1038/s41467-020-16969-0 10.1038/s41467-020-16969-0 PMC732001032591531

# Peer review: Reviewer response for version 2

**DOI:** 10.21956/wellcomeopenres.18307.r42581

**Published:** 2021-02-23

**Authors:** Michael Chong, Pedrum Mohammadi-Shemirani

**Affiliations:** 1 Population Health Research Institute (PHRI), David Braley Cardiac, Vascular and Stroke Research Institute, Hamilton Health Sciences, Hamilton, ON, Canada; 2 Thrombosis and Atherosclerosis Research Institute, Hamilton, ON, Canada; 3 Department of Pathology and Molecular Medicine, Michael G. DeGroote School of Medicine, McMaster University, Hamilton, ON, Canada; 4 Population Health Research Institute (PHRI), David Braley Cardiac, Vascular and Stroke Research Institute, Hamilton Health Sciences, Hamilton, ON, Canada; 5 Department of Biochemistry and Biomedical Sciences, McMaster University, Hamilton, ON, Canada

Firstly, I thank the Wellcome Open Research editorial team for the opportunity to provide feedback on the review by Gill *et al.* (2021), entitled “Mendelian randomization for studying the effects of perturbing drug targets”. I have reviewed this work in detail in concert with Pedrum Mohammadi-Shemirani and the subsequent comments reflect our joint assessment. Gill *et al.* reviewed an increasingly popular application of Mendelian Randomization analyses to understand causal on-target effects of drug targets on disease risk. Current practices, considerations, and limitations of this approach were thoroughly summarized and thus we only provide minor comments. Firstly, while Figure 1 is a useful illustration, the last panel may be misinterpreted as decreasing LDL is associated with a greater risk of CAD. As such, Figure 1 should be amended for clarity. Secondly, as authors correctly acknowledge, restricting instruments to cis variation is the standard practice to mitigate the potential for genetic confounding, but it should also be noted that this still leaves open the possibility for genetic confounding, particularly in regions containing multiple protein-coding genes. Multivariable MR conditioning for the effects of the genetic instruments on the surrounding gene products may represent a way to assess whether findings may be confounded by proximal gene products when such data is available, an approach implemented by Porcu *et al.* (2019) ( https://www.nature.com/articles/s41467-019-10936-0). ^1^ Similarly, in the section titled “Selecting genetic variants to proxy drug targets”, a qualifying statement may be warranted regarding the selection of genetic variants based on association with known indications. If pQTL data is available, this approach may better serve instrument validation while a selection of variants is supported by evidence of association with protein expression or function. If no such data is available, evidence of association with known indications can be used, but it warrants a cautious approach with careful consideration of the *cis* window and other protein-coding genes therein. Thirdly, authors should further comment on the careful use of trans-instruments to approximate drug targets as was executed by Georgakis *et al.* (2019) https://pubmed.ncbi.nlm.nih.gov/30586705/. ^2^ Fourth, we appreciate the authors’ decision to include example resources and databases to guide instrument selection (e.g., Ensembl, DrugBank, etc.), particularly for novice readers. Would the authors be able to provide similar suggested resources for other sections? For instance, databases to identify promoter and enhancer regions (e.g., GeneCards), or tools to help investigate violations of the independence and exclusion restriction assumptions (e.g., PhenoScanner). Lastly, we believe the following statement illustrates a very important point: “ *Moreover, comparing instruments between different MR studies of the same exposure can provide additional evidence. For example, both Gill et al. and Walker et al. independently derived instruments for antihypertensive drug effects that perform comparably when tested against a common outcome* *^44^* *^,^* *^55^* ** *Although different MR studies may use similar or overlapping data sources, different instrument selection approaches can make analyses vulnerable to distinct biases and so also have a role in triangulation of evidence.*” Perhaps this should be briefly commented on in the context of technological biases between different proteomics and metabolomic technologies (for example https://www.ncbi.nlm.nih.gov/pmc/articles/PMC6277005/) ^3^ thus further emphasizing the importance of ensuring the robustness of MR results using alternative instruments. As proteome-wide pQTL data becomes increasingly available to the public, such sensitivity analyses should become more commonplace. Overall, this review is exquisitely written, thoughtful, and appropriately referenced. Accordingly, we have no major concerns and only offer the aforementioned minor comments.

Is the review written in accessible language?

Yes

Are all factual statements correct and adequately supported by citations?

Yes

Are the conclusions drawn appropriate in the context of the current research literature?

Yes

Is the topic of the review discussed comprehensively in the context of the current literature?

Yes

Reviewer Expertise:

Human genetics, genetic epidemiology

We confirm that we have read this submission and believe that we have an appropriate level of expertise to confirm that it is of an acceptable scientific standard.

## References

[1] : Mendelian randomization integrating GWAS and eQTL data reveals genetic determinants of complex and clinical traits. *Nature Communications*.2019;10(1) : 10.1038/s41467-019-10936-0 10.1038/s41467-019-10936-0 PMC665677831341166

[2] : Genetically Determined Levels of Circulating Cytokines and Risk of Stroke. *Circulation*.2019;139(2) : 10.1161/CIRCULATIONAHA.118.035905 256-268 10.1161/CIRCULATIONAHA.118.035905 30586705PMC7477819

[3] : In Aptamers They Trust. *Circulation*.2018;138(22) : 10.1161/CIRCULATIONAHA.118.036823 2482-2485 10.1161/CIRCULATIONAHA.118.036823 30524136PMC6277005
